# Study on High-Temperature Activated Products and Hydration Properties of Aga Soil in Tibet for Cement Concrete

**DOI:** 10.3390/ma17215364

**Published:** 2024-11-01

**Authors:** Lihui Li, Kaiming Niu, Jianrui Ji, Panpan Zhang, Jilin Zhang

**Affiliations:** 1 School of Civil Engineering, Chongqing Jiaotong University, Chongqing 400074, China; km.niu@rioh.cn (K.N.); 18894009807@163.com (J.J.); 15223123773@139.com (J.Z.); 2 Institute of Highway Science, Ministry of Transport, Beijing 100088, China; zhangpanpan648@126.com

**Keywords:** Aga soil, high-temperature calcination furnace, water-hardened cementitious materials, calcination products, heat of hydration

## Abstract

In order to impart the properties of cementitious material to the Tibetan Agar soil, two high-temperature activation mechanisms (HTMA, HTMB) were designed in this study, and the products and hydration-hardening properties of Tibetan Agar soil high-temperature activation mechanism were analyzed by means of SEM, XRD, and XRF. The results show that the main components of Tibetan Aga soil are calcite and quartz; Aga soil is activated by HTMA high-temperature activation, forming the main products of CaO, C_2_S, CaSiO_3_, and CaAl_2_Si_2_O_8_, and its products have both air-hardening and water-hardening characteristics; Aga soil is activated by HTMB high-temperature activation, and when the temperature reaches 1250 °C when the clinker is not found in the CaO, the generation of C_2_S, C_3_S, C_3_A, C_4_AF, and Mg_2_SiO_4_ minerals with good water-hardening cementitious properties occurs when the temperature rises to 1350 °C, although the formation of some inert minerals that do not have the cementitious properties, but this temperature activation products of the thermodynamic properties of the best; Enhancing the value of lime saturation degree (KH) and silicon rate (SM) can promote the formation of the products of the C_2_S and C_3_S, increase the reactivity of the Aga soil activation products, and increase the hydration heat as well as compressive and flexural strength, combined with the results of the hydration heat and mechanical test, KH is recommended to be 0.9~0.94, SM is recommended to be 1.8~2.4, and alumina ratio (IM) is recommended to be 1.8~2.4 when Aga soil is used with raw materials.

## 1. Introduction

Aga soil is a unique natural mineral in Tibet, mainly distributed in the middle valley of the Yarlung Zangbo River [1]. The Tibetan people have long used Aga soil as an important building material. It is usually combined with water and mud and is applied to Tibetan architecture in the way of “playing aga”. However, the strength of Aga soil material is low, the waterproof performance is poor, the internal cementing material is easily washed away by rainwater, and it becomes rough and cracked under the weather conditions of sunshine, rain, wind erosion, and freeze-thaw. Aga soil does not have cementing properties, but after high-temperature activation, it has the dual properties of “hydraulic set” and “air setting”. Xinjiang also has a soil composition similar to aga soil composition, known as “ginger nuts” [2].

At present, mainly by combining Aga soil with other substances such as silicone coatings [3], PS main agent [4], fly ash, and other substances after modification, Aga soil is applied to the restoration of cultural relics, increasing the waterproof performance of Aga soil slabs and roofs, etc. In ancient times, it was also applied to buildings by combining Aga soil with elm wood sap and sandstone. Li li [5] carried out high-temperature modification of natural Aga soil and ginger nuts at 1000 °C and found that after high-temperature modification, it had the basic properties of small shrinkage deformation, large porosity, good water permeability, and air permeability [6,7,8].

In foreign countries, this kind of ore with certain cementitious properties after direct high-temperature activation is called “natural hydraulic lime” [9]. García [10] used the suspension of bioproducts obtained from the fermentation of biodiesel crude glycerol to prepare natural hydraulic lime mortar, which improved the mechanical properties and reduced the water absorption of the mortar. James [11] added two modifiers, and the compressive strength reached 200% of the traditional lime mortar. Wang [12] and Zhang [13] tried to add milk and egg white to natural hydraulic lime, and the egg white could substantially increase the compressive strength of lime at the age of 28 d by 176% compared to the control sample. Cechova [14] found that adding 1~3 wt.% of flaxseed oil to natural hydraulic lime mortar could improve the waterproofness of lime mortar. Silva [15] found that 50% natural hydraulic lime mixed mortar is more suitable for cultural relic restoration.

The above scholars have put forward many methods to optimize the material properties for this kind of lime, but there are few studies on optimizing the properties of this material from the perspective of high-temperature activation. Thus, in order to improve the gelling activity of Agar soil, this study explored the changing law of the products of Agar soil at high temperatures through different high-temperature activation mechanisms and proposed a high-temperature modification method, which provides a theoretical basis for the application of Agar soil in Tibet.

## 2. Materials and Methods

### 2.1. Raw Materials

#### 2.1.1. Natural Aga Soil

The natural Aga soil is taken from Zhanan County, Shannan City, Tibet Autonomous Region, and its chemical composition is shown in Table 1.

#### 2.1.2. Chemical Reagents

The clay XRF oxide content used in the test is shown in Table 2, and analytically pure SiO_2_, Fe_2_O_3_, and CaSO_4_ are from Shanghai Aladdin Chemical Reagent Company.

### 2.2. Methods

#### 2.2.1. High-Temperature Activation Method

**Figure 1:**
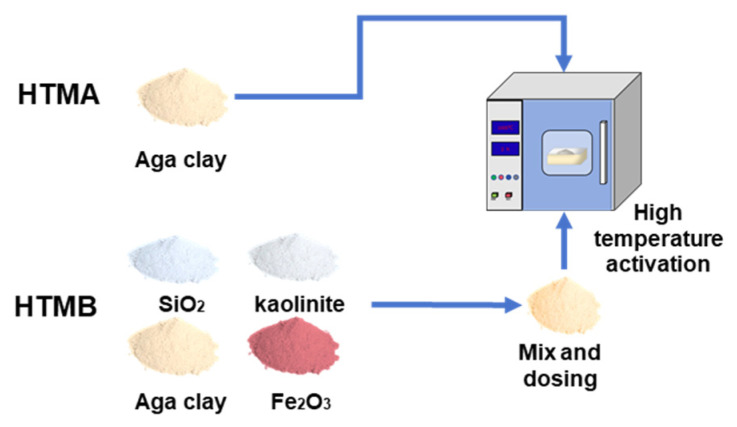
High-temperature activation diagram.

**Figure 2:**
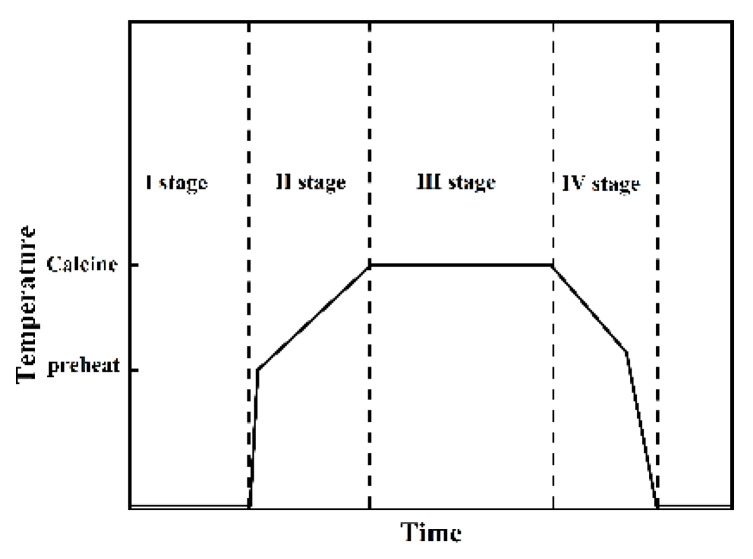
Mechanism of high-temperature activation temperature of Aga soil.

To explore the pyrolysis characteristics and product changes of natural Aga soil minerals under different high-temperature activation mechanisms. As shown in Figure 1, two kinds of high-temperature activation methods were used to study the aga soil, namely HTMA and HTMB. The thermodynamic temperature variation pattern of Aga soil with different high-temperature activation methods [16] is shown in Figure 2. Two kinds of high-temperature activation methods are divided into four stages. The first stage is the furnace preheating stage; HTMA and HTMB are preheated to 800 °C and 950 °C, respectively. The second stage is the specimen heating. The specimen is put into the high-temperature activation furnace and then heated up rapidly, and then with the high-temperature activation furnace synchronous heating. Stage III is the specimen constant temperature; HTMA and HTMB for 120 min and 45 min, respectively. The fourth stage is cooling. In order to protect the high-temperature activation furnace components and ensure the safety of the test, the specimen is first cooled to 900 °C synchronously with the high-temperature activation furnace, and then the specimen is taken out and cooled to room temperature.

HTMA: between 800 °C and 1400 °C, with 50 °C as the temperature gradient. The corundum crucible with a specification of 295 mm × 195 mm × 75 mm is used to carry out the high-temperature activation test by fixedly weighing 2 kg of Aga soil powder each time. HTMB: by adding clay, SiO_2_, Fe_2_O_3_ natural minerals or analytically pure substances, adjust the lime saturation degree (KH), silicon rate (SM), and aluminum rate (IM), the test adjustment scheme is shown in Table 3, the calculation unit is wt.%, and the calculation method is shown in Equations (1)~(3). Each protocol is proportioned at 100 g mass. After thorough mixing, it was put into a 200 mL corundum crucible for the high-temperature activation test.


(1) $$KH=\frac{wt.\%(CaO)-1.65\times {wt.\%(Al}_{2}O_{3})-0.35\times {wt.\%(Fe}_{2}O_{3})}{2.8\times {wt.\%(SiO}_{2})}$$

(2) $$SM=\frac{{wt.\%(SiO}_{2})}{wt.\%({Al}_{2}O_{3})+wt.\%({Fe}_{2}O_{3})}$$

(3) $$IM=\frac{{wt.\%(Al}_{2}O_{3})}{{wt.\%(Fe}_{2}O_{3})}$$

#### 2.2.2. Test Methods

Hydration heat test: The 72 h hydration heat release of Aga soil after high-temperature activation was measured by Thermometri TAM AIR eight-channel microcalorimeter. Before the hydration heat test, 2.5 wt.% CaSO_4_·2H_2_O was added to each group of samples and mixed evenly. Each group of samples was taken at 10 g, and 4.5 g of pure water was added; that is, the water–cement ratio was 0.45, and 6.305 g of reference water was added to the corresponding control channel.

Mechanical performance test: Implemented in accordance with the provisions of T 0506 in the “Highway Engineering Cement and Cement Concrete Test Specification” (JTG 3420-2020) [17]. The cement mortar test block was made of 225 g of cement, 225 g of Aga soil, 1350 g of standard sand, and 225 mL of water to test the flexural compressive strength of hydrated 3 d, 7 d, and 14 d ages. There are three mortar specimens in each group, and the average value is taken as the final test value after testing.

Hydration product test: The high-temperature activated Aga soil powder was made into 40 mm × 40 mm × 40 mm at a water-solid ratio of 0.5 and cured to 1 d, 7 d, and 14 d of age for XRD and SEM tests.

## 3. Results and Discussions

### 3.1. HTMA

**Figure 3:**
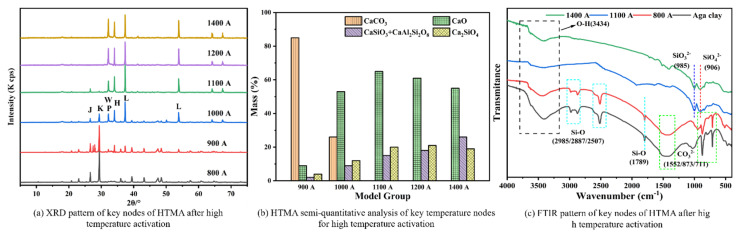
HTMA product variation diagram. Note: 800 A represents HTMA, 800 °C high-temperature activated Aga soil; J-SiO_2_, K-CaCO_3_, W-CaSiO_3_, P-CaAl_2_Si_2_O_8_, L-CaO, and H-Ca_2_SiO_4_.

In this experiment, the Ca:Si of Aga soil used is 5.7, Ca:Al is 17.9, and Si:Al is 3.2, and according to the XRD results of Figure 3, the calcium-containing compounds in Aga soil were analyzed semi-quantitatively, and the results are shown in Figure 3b. The results of IR diffraction analysis in Figure 3c are consistent with XRD. It can be seen from Figure 3a that under the high-temperature activation of HTMA, Aga soil formed four new products, namely CaO, CaSiO_3_, Ca_2_SiO_4_ (hereinafter referred to as C_2_S [18], and CaAl_2_Si_2_O_8_, which is in agreement with the results of the study by Linyi Zhao [5]. A small amount of CaO was found at 800 A, indicating a small amount of calcite decomposition; at 900 A, the CaO diffraction peaks turned strong, while new products CaSiO_3_, C_2_S, and CaAl_2_Si_2_O_8_ appeared, and there was a change in the composition of the SiO2 diffraction peaks, which indicated that the crystal transformation of SiO_2_ to α-tridymite occurred [19]; at 1000 A, the CaO diffraction peaks were sharp, and the diffraction peaks of the new products CaSiO_3_, C_2_S, and CaAl_2_Si_2_O_8_ turned strong, at which time, the diffraction peaks of calcite and quartz in the raw ore of Aga soil became weaker; at 1100 A, the diffraction peak of calcite disappeared completely and the CaO diffraction peak reached the strongest, while calcite decomposition is a reversible chemical reaction [20], indicating that at 1100 °C, the transformation became a sufficient chemical reaction; at 1200 A, the diffraction peaks of SiO_2_ disappeared, meanwhile, the diffraction peaks of CaO began to weaken, and the diffraction peaks of CaSiO_3_, C_2_S, and CaAl_2_Si_2_O_8_ continued to enhance, indicating that all of SiO_2_ reacted to CaSiO_3_, C_2_S, and CaAl_2_Si_2_O_8_ at 1200 °C; at 1400 A, the diffraction peaks of CaO were weakened, and the diffraction peaks of CaSiO_3_ and CaAl_2_Si_2_O_8_ were enhanced. In the range of 1200 °C~1400 °C, the XRD diffraction peaks were all new products formed by the high-temperature reaction of Aga soil, but the diffraction peaks of each new product at different temperatures had obvious changes, which indicated that CaSiO_3_, CaAl_2_Si_2_O_8_, and CaO were transforming, and with the increase in temperature, CaO and CaAl_2_Si_2_O_8_ decreased and CaSiO_3_ increased.

**Figure 4:**
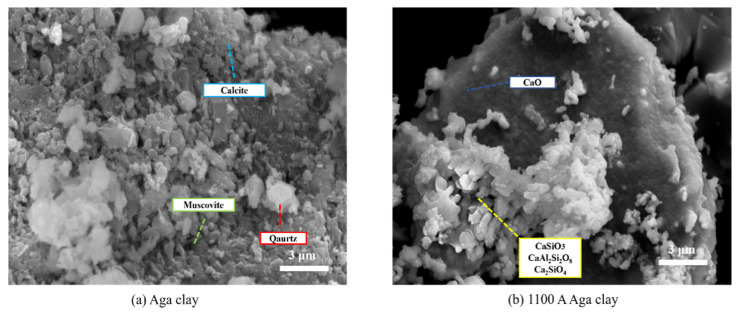
Natural Aga soil and 1100 A SEM.

Figure 4 shows the scanning electron microscope images of natural Aga soil, and 1100 A Aga soil shows the changes of products in Aga soil. At 1100 A, a large amount of calcite decomposes into CaO, which is small molecules and clusters together, and some of the CaSiO_3_, C_2_S, and CaAl_2_Si_2_O_8_ formed by reaction with quartz are distributed on the surface of CaO.

The mass ratio of calcite (CaCO_3_) in Aga soil is more than 85%, and the high-temperature activation process is dominated by the transformation between calcium-containing compounds. Calcite (CaCO_3_) in Aga soil gradually decomposed into CaO with the increase in temperature, and it was completely decomposed at 1100 °C. At the same time, the CaO content with the increase in temperature first increased and then decreased, with the highest content at 1100 °C, which is related to the decomposition of CaCO_3_ and the rate of reaction between CaO and SiO_2_ [21]. As the reaction between CaO with SiO_2_ and Al_2_O_3_ continues, the yields of the reaction products C_2_S and CaSiO_3_+ CaAl_2_Si_2_O_8_ gradually increase. The chemical reactions occurring in Aga soil at specific high temperatures are as follows:

800 °C~1200 °C, CaCO_3_ decomposes, and new products form as follows:

*CaCO*_3_ → *CaO* + *CO*_2_↑ (4)

2 *CaO* + *SiO*_2_ → *Ca*_2_*SiO*_4_ (5)

*CaO* + *SiO*_2_ → *CaSiO*_3_ (6)

*CaO* + *Al*_2_O_3_ + 2 *SiO*_2_ → *CaAl*_2_*Si*_2_*O*_8_ (7)

1200~1400 °C, reactions between new products:

*CaAl*_2_*Si*_2_*O*_8_ + *CaO* → 2 *CaSiO*_3_ + *Al*_2_*O*_3_ (8)

### 3.2. HTMB

#### 3.2.1. XRD

(H - C_2_S, S - 3CaO·SiO_2_, N - 3CaO·Al_2_O_3_, M - 4CaO·Al_2_O_3_·Fe_2_O_3_, I - Mg_2_SiO_4_, L - CaO)

**Figure 5:**
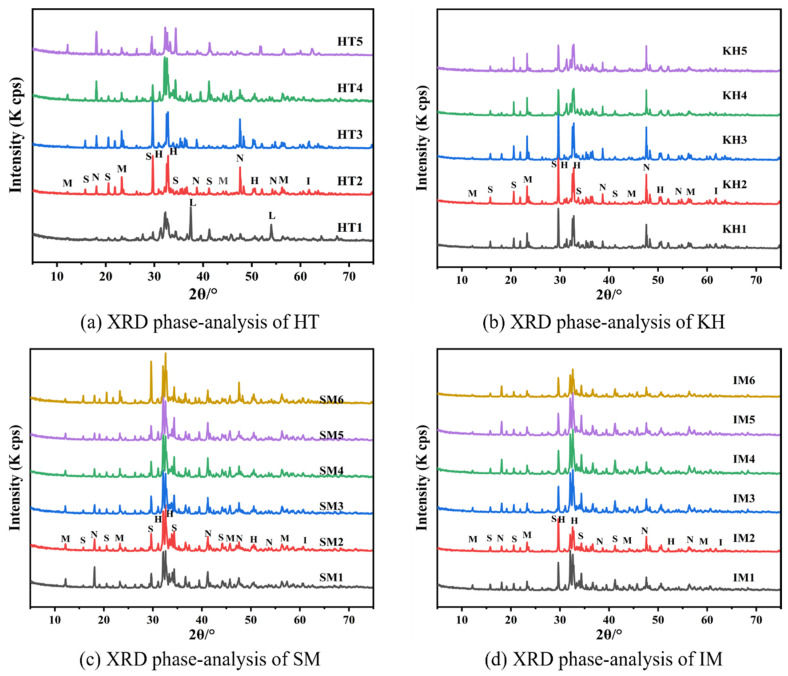
HTMB XRD pattern of each scheme.

Figure 5 shows the XRD fluorescence diffraction pattern after HTMB high-temperature activation according to the scheme of Table 3, which observed C_2_S, 3CaO-SiO_2_ (C_3_S), 3CaO-Al_2_O_3_ (C_3_A), 4CaO-Al_2_O_3_-Fe_2_O_3_ (C_4_AF), and Mg_2_SiO_4_, but it is noteworthy that the diffraction peaks of the substances CaSiO_3_ and CaAl_2_Si_2_O_8_ produced under HTMA were not detected, which may be related to the KH, SM, and IM parameters in the Aga soil system.

It can be seen from Figure 5a that when the high-temperature activation temperature reaches 1200 °C, a strong CaO diffraction peak is detected in the spectrum, which indicates that there is a large amount of CaO in the system. It must be noted that when the high-temperature activation temperature reaches or exceeds 1250 °C, the diffraction peak of CaO disappears, which indicates that 1250 °C is the critical temperature for the full chemical reaction of CaO with SiO_2_, Al_2_O_3_, and Fe_2_O_3_ in Aga soil. In addition, the comparison of the plots observed that the accompanying increase in temperature can promote the formation of C_3_A and C_4_AF, but when the temperature reaches 1400 °C, it instead causes a small weakening of the C_3_S and C_2_S peaks, and there are high caking hardness, difficult to grind, a large number of holes, and many impurities after high-temperature activation. Therefore, under HTMB, 1350 °C is the best high-temperature activation temperature for the test group.

Figure 5b–d, along with the changes of KH, SM, and IM, there is a small change in the intensity of the diffraction peaks of the main cementitious products formed, C_2_S, C_3_S, C_3_A, and C_4_AF. The reason is that KH, SM, and IM change the proportion of Ca, Si, Al, and Fe elements in the Aga soil system. Specifically, when Ca/Si = 5.7 in the Aga soil system, the high-temperature activation products contain CaO, C_2_S, CaAl_2_Si_2_O_8_, and CaSiO_3_; when Ca/Si = 3.0~3.8, Al/Fe = 1.2~2.7, and the high-temperature activation temperature exceeds 1250 °C, the high-temperature activation products are transformed into C_2_S, C_3_S, C_3_A, and C_4_AF with good water-hardening properties. The XRD test results of Figure 5 show that KH should be 0.9~0.94, SM should be 1.8~2.4, and IM should be 1.8~2.4 when calculating the raw materials.

#### 3.2.2. Hydration Heat

**Figure 6:**
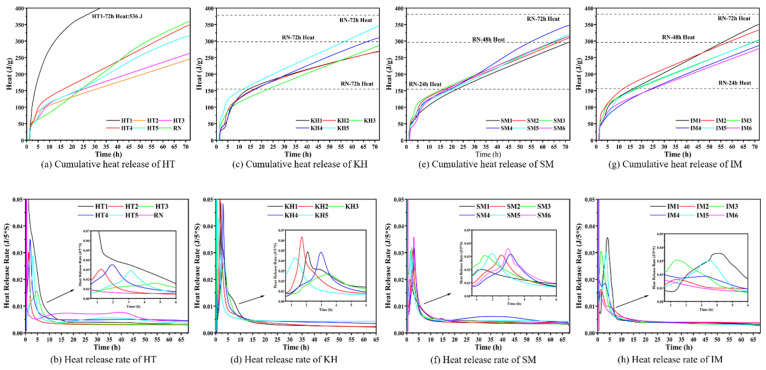
The cumulative heat of hydration and heat release rate of each HTMB scheme in 3 days.

According to the above XRD analysis results, it is shown that the main products of Aga soil after high-temperature activation under HTMB have good hydraulicity. We tested the 3 d hydration heat of each group according to the scheme in Table 3. The test results are shown in Figure 6, where RN is the reference group and the material is P•I 42.5 reference cement.

Observing Figure 6a,c,e,g, it can be seen that the test groups except for HT1, compared with RN, showed a fast hydration rate in the early stage and a low hydration rate in the late stage, with the main demarcation time at 20 h~40 h. In addition, the cumulative hydration heat of the test group for 3 days never exceeded the RN. Within 5 h of hydration time, sudden changes in hydration rate occurred in all test groups, which were related to the small amount of calcium, aluminum, iron oxide impurities, and gypsum content in the test group. The 3 d cumulative hydration heat of HT1 in the test group was significantly higher than that in the other groups because HT1 contained a large amount of CaO and its hydration exotherm was accelerated after overcooking [22]; HT2~HT5, comprehensively view HT4, i.e., the high-temperature activation temperature of 1350 °C is the closest to RN. Figure 6c, KH1~KH5, 3 d cumulative heat release increases with the increase in KH, because C_3_A and C_3_S also increase with the increase in KH, and the hydration heat release of C_3_A and C_3_S is higher than that of C_2_S [23]. Figure 6e, SM1~SM6, with the increase in silicon rate, 3 d cumulative heat release first increased and then decreased. In Figure 6g, IM1~IM6, the cumulative heat release decreases with the increase in the aluminum ratio. Although the increase in aluminum ratio increases the formation of C_3_A, C_3_S, and C_4_AF, a decrease, especially the decrease in C_3_S, leads to the decrease in hydration heat.

It can be seen from Figure 6b,d,f,h that RN clearly shows the initial hydration period, induction period, hydration acceleration period, and hydration deceleration period of reference cement hydration [24]. The exothermic rate of the test group during the initial hydration period was almost the same as that of the RN group, but the exothermic rate of the test group during the other periods was significantly different from that of the RN group, focusing on the induction and acceleration periods, with the duration of the induction period of the test group being only 1~1.7 h while that of the RN amounted to 4 h. The acceleration period is also the same, and the length of the acceleration period in the test group is only 6.2~8.2 h while the RN reaches 20 h, which indicates the low workability of the hydrated slurry after the high-temperature activation of the Aga soil HTMB. It is inferred that the main reason is the low gypsum content of the test group, as well as the insufficient grinding of calcium, aluminum, and iron oxide impurities, which make the mixing between the components uneven, leading to the rapid hydration reaction of C_3_A, C_3_S, C_2_S, and C_4_AF and shortening the hydration induction and accelerated period.

### 3.3. Mechanical Properties and Hydration Products

#### 3.3.1. Mechanical Strength

**Figure 7:**
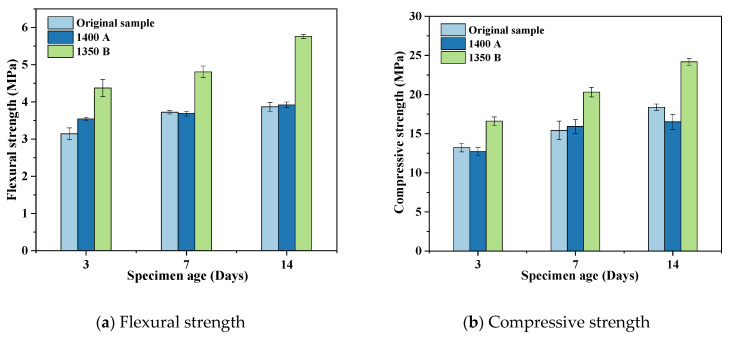
50% cement replacement ratio 3 days, 7 days, 14 days mechanical strength. (Natural Aga soil, 1400 A, and 1350 B are represented by AO, AA, and AB, respectively).

Figure 7 shows the 3 d, 7 d, and 14 d flexural compressive strengths of cement mortar test blocks made at a 50% cement replacement rate.

It can be seen from Figure 7a that the flexural strength of the AO group only increases slowly with the increase in age because the cementitious components in the test block are greatly reduced under high replacement rates. The flexural strength of the AA group increases with age, and the rate of increase is slower than that of the AO group, but the flexural strength is slightly higher than that of the AO group. The reason is that 1400 A contains a large amount of CaO, and the product formed after the hydration of CaO has poor gelling properties and causes volumetric expansion, which makes the AA group, although higher than AO in terms of gelling components, the actual effect is not the same. The flexural strength of AB increases significantly with age, and the flexural strength is significantly higher than that of AO and AA groups, but there is still some gap compared with the reference cement. The former is because the AB has good cementitious components, and the latter is that the AB group contains a certain amount of impurities. Figure 7b, the compressive strength test results are similar to the flexural strength; the difference is that the compressive strength of the AA group is lower than that of the AO group.

#### 3.3.2. SEM and XRD of Hydration Products of HTMB Aga Soil Paste

**Figure 8:**
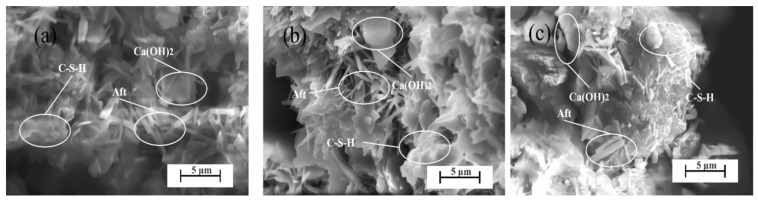
SEM of hydration products of HTMB Aga soil pure slurry at (**a**) 1 d, (**b**) 3 d, and (**c**) 14 d.

**Figure 9:**
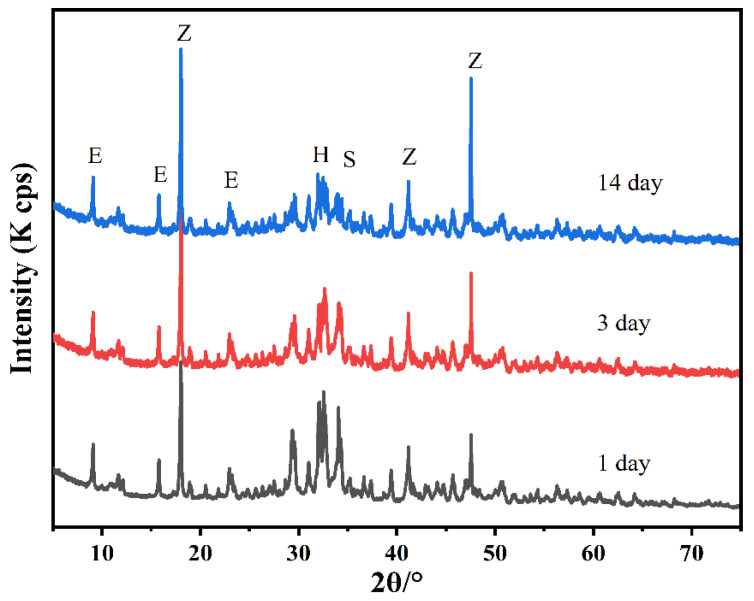
XRD of hydration products of HTMB Aga soil pure slurry at 1 d, 3 d, and 14 d.

Observing Figure 8a–c, there are hexagonal crystals [25], particulate crystals [26], and needle-like cubic crystals [27] in the SEM image. Combined with Figure 9, the three crystals are Ca(OH)_2_, C-S-H gel, and AFt, and the length of AFt reaches 3 μm. The three kinds of hydration products have complete crystals and are well formed, and the C-S-H gel continues to increase with the increase in hydration time, but the hydration products are not sufficiently full of each other, which explains that in the case of HTMB with good cementitious components, the flexural and compressive strength of the AB group still has a large gap with the strength of ordinary silicate cement.

## 4. Conclusions

(1) When HTMA high-temperature activation is used, Tibetan Aga soil produces activation products such as CaO, C_2_S, CaSiO_3_, CaAl_2_Si_2_O_8_, etc. Calcite in Aga soil is completely decomposed at 1100 °C and forms CaO and CaAl2Si2O8 minerals, of which the CaO content increases and then decreases with increasing temperature, and the content is highest at 1100 °C, while the content of C_2_S, CaSiO_3_, and CaAl_2_Si_2_O_8_ products increases with increasing temperature. From the CaO, C_2_S, CaSiO_3_, and CaAl_2_Si_2_O_8_ products, it can be illustrated that the Aga soil activated with HTMA at high temperatures has both air hardening and hydraulicity characteristics.

(2) When HTMB is activated at high temperature when the temperature reaches 1250 °C, there is no CaO component in the activated clinker, and C_2_S, C_3_S, C_3_A, C_4_AF, Mg_2_SiO_4,_ and other minerals with good hydraulic gelation properties are formed by the reaction. However, CaSiO_3_ and CaAl_2_Si_2_O_8_ minerals produced under HTMA are not found, and with the increase in activation temperature to 1350 °C, Ca_2_Fe_2_O_5_, Ca_2_AlFeO_5_, Ca_2_Fe_7_O_11_, Ca_4_Fe_14_O_25_, Ca_12_Al_14_O_33_, CaFeO_3,_ and other inert minerals with stable structure and no gelation were produced.

(3) Due to the high content of CaO, the HTMA-activated Aga soil is not conducive to the development of the hydraulic strength of the product. By using the HTMB activation mechanism to increase the KH and SM values, the formation of C_2_S and C_3_S products can be promoted, and the hydration reactivity, hydration heat, and compressive and flexural strength of the activated products of Aga soil can be improved. Combined with the results of the hydration heat and mechanical test, KH is recommended to be 0.9~0.94, SM is recommended to be 1.8~2.4, and IM is recommended to be 1.8~2.4 when Aga soil is mixed with raw materials.

## Figures and Tables

**Table 1:** XRF oxide content in Aga soil.

Chemical Composition	CaO	SiO_2_	Al_2_O_3_	Fe_2_O_3_	MgO	SO_3_	K_2_O	P_2_O_5_	N_2_O	LOI
(w%)	44.87	8.50	2.28	1.14	1.00	0.07	0.41	0.05	0.21	41.19

**Table 2:** XRF oxide content of clay.

Chemical Composition	Al_2_O_3_	SiO_2_	P_2_O_5_	SO_3_	K_2_O	CaO	TiO_2_	Fe_2_O_3_	SrO	ZrO_2_
(w%)	40.65	57.66	0.09	0.45	0.23	0.06	0.48	0.30	0.02	0.02

**Table 3:** High-temperature activation HTMB scheme.

Group	T/(°C)	KH	SM	IM
HT1~HT5	1200~1400	0.92	1.8	1.5
KH1~KH5	1350	0.86~0.94	1.8	1.5
SM1~SM6	1350	0.92	0.9~2.4	1.5
IM1~IM6	1350	0.92	1.8	1.2~2.7

## Data Availability

The original contributions presented in the study are included in the article, further inquiries can be directed to the corresponding author.
